# Reduced PAK1 activity sensitizes FA/BRCA-proficient breast cancer cells to PARP inhibition

**DOI:** 10.18632/oncotarget.12576

**Published:** 2016-10-11

**Authors:** Olga Villamar Cruz, Tatiana Y. Prudnikova, Daniela Araiza-Olivera, Carlos Perez-Plasencia, Neil Johnson, Andrea J. Bernhardy, Michael Slifker, Catherine Renner, Jonathan Chernoff, Luis E. Arias

**Affiliations:** ^1^ UBIMED, Facultad de Estudios Superiores-Iztacala, UNAM, Tlalnepantla, Estado de México, Mexico; ^2^ Cancer Biology Program, Fox Chase Cancer Center, Philadelphia, PA, USA; ^3^ Experimental Therapeutics Program, Fox Chase Cancer Center, Philadelphia, PA, USA; ^4^ Department of Biostatistics and Bioinformatics, Fox Chase Cancer Center, Philadelphia, PA, USA; ^5^ Department of Pathology, Fox Chase Cancer Center, Philadelphia, PA, USA

**Keywords:** transformation, PAK1, DNA repair, Fanconi Anemia, small molecule inhibitor

## Abstract

Cells that are deficient in homologous recombination, such as those that have mutations in any of the Fanconi Anemia (FA)/BRCA genes, are hypersensitive to inhibition of poly(ADP-ribose) polymerase (PARP). However, FA/BRCA-deficient tumors represent a small fraction of breast cancers, which might restrict the therapeutic utility of PARP inhibitor monotherapy. The gene encoding the serine-threonine protein kinase p21-activated kinase 1 (PAK1) is amplified and/or overexpressed in several human cancer types including 25-30% of breast tumors. This enzyme controls many cellular processes by phosphorylating both cytoplasmic and nuclear substrates. Here, we show that depletion or pharmacological inhibition of PAK1 down-regulated the expression of genes involved in the FA/BRCA pathway and compromised the ability of cells to repair DNA by Homologous Recombination (HR), promoting apoptosis and reducing colony formation. Combined inhibition of PAK1 and PARP in PAK1 overexpressing breast cancer cells had a synergistic effect, enhancing apoptosis, suppressing colony formation, and delaying tumor growth in a xenograft setting. Because reduced PAK1 activity impaired FA/BRCA function, inhibition of this kinase in *PAK1* amplified and/or overexpressing breast cancer cells represents a plausible strategy for expanding the utility of PARP inhibitors to FA/BRCA-proficient cancers.

## INTRODUCTION

p21-activated kinases (PAKs) are effectors for the small GTPases Cdc42 and Rac that control several cellular processes, including cell morphology, motility, survival, gene transcription, apoptosis and hormone signaling [1-3]. These enzymes are widely expressed in numerous tissues and are activated by extracellular signals through GTPase-dependent and -independent mechanisms [4]. In addition, it has been shown that a gene encoding one member of the PAK family, PAK1, located on human chromosome 11q13, is amplified and/or overexpressed in several human cancer types, including 25-30% of breast tumor samples and cancer cell lines [3]. In addition to its well-characterized kinase activity, it is documented that PAK1 translocates into the nucleus and associates with chromatin, suggesting that it might be involved in gene transcription [5, 6]. More recently, PAK1 signaling has emerged as a component of the DNA damage response as PAK1 activity influences the cellular sensitivity to ionizing radiation [7, 8].

When the DNA is damaged, it is repaired by two different mechanisms. PARP is involved in the repair of DNA single-strand breaks (SSBs), and when it is inhibited, DNA SSBs degenerate to more lethal DNA double-strand breaks (DSBs) that require repair by homologous recombination (HR), which requires the activation of the Fanconi Anemia (FA)/BRCA pathway, a DNA-damage response signaling pathway which is essential for the repair of DNA interstrand cross-links induced by DNA-damaging agents [9-11]. Therefore, FA/BRCA-deficient cells and other cells that are defective in homologous recombination are highly susceptible to poly(ADP-ribose) polymerase (PARP) inhibition [12, 13].

Here, we show that some genes involved in the FA/BRCA pathway are down-regulated in PAK1 deficient cells. The expression of two FA genes, FANCD2 and FANCI, was confirmed by qPCR and western blot in PAK1 depleted human breast cancer cells with or without *PAK1* amplification and/or overexpression. Interestingly, the depletion or chemical inhibition of PAK in *PAK1* amplified or overexpressing breast cancer cells treated with DNA damaging agents, compromised the ability of these cells to form Rad51 foci, induced cell cycle arrest, promoted apoptosis and resulted in reduced colony formation. In contrast, the inhibition or depletion of PAK1 had little effect on these cellular processes in *PAK1*-non-amplified breast cancer cells*.* Finally, we showed that combined inhibition of PAK and PARP had a synergistic effect in *PAK1* amplified or overexpressing breast cancer cells, were the dual inhibition of these molecules totally abrogated colony formation, enhanced apoptosis and impaired tumor growth in a xenograft setting. Interestingly, the ectopic overexpression of PAK1 in *PAK1*-non-amplified breast cancer cells recapitulated the sensitivity to combined inhibition of PAK and PARP observed in *PAK1*-amplified breast cancer cells, suggesting that PAK1 is involved in DNA repair by HR through a FA/BRCA dependent pathway. These findings indicate that depletion or inhibition of PAK1 creates a state of “FA/BRCAness” in transformed cells and represents a rational approach for expanding the utility of PARP inhibitors to FA/BRCA-proficient cancers.

## RESULTS

### Fanconi anemia genes are down-regulated in PAK1 deficient Cells

**Figure 1:**
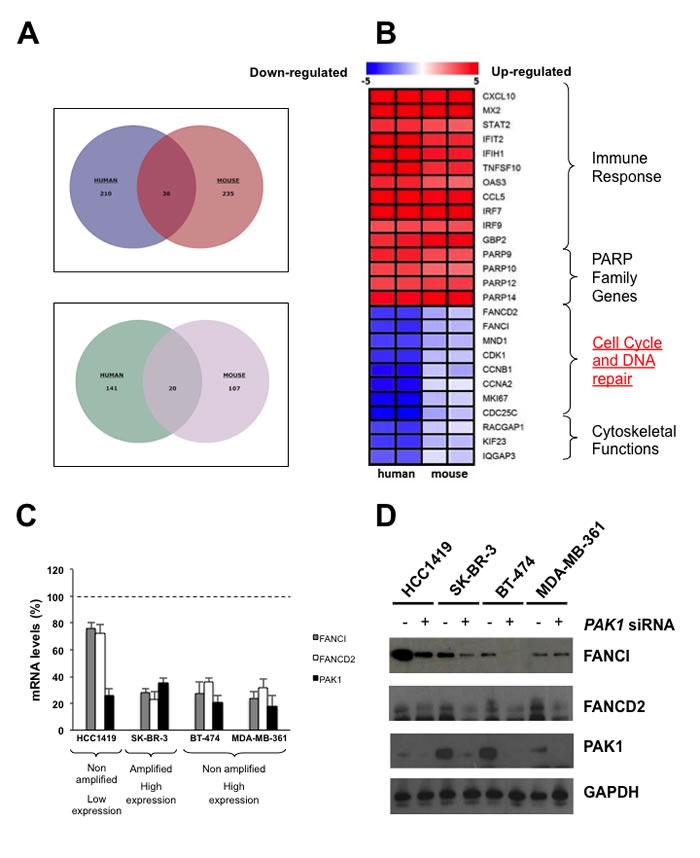
PAK inhibition down-regulates FA genes **A.** Venn diagrams showing differentially regulated genes between wild-type and PAK1 deficient mouse and human breast cancer cells. The upper panel shows the up-regulated genes and the lower panel the down-regulated genes. **B.** Heat map representation of microarray data. Genes were classified according to their cellular function. The color scale represents the expression level of a gene above (red) or below (blue) the mean expression levels across the samples. **C.** qPCR validation of microarray data. PAK1-overexpressing and/or amplified, and non-amplified breast cancer cells were transfected with non-targeting or *PAK1* targeting siRNAs. All data were normalized to control GAPDH. Fold changes were calculated using the ΔCt method (2^−ΔΔCt^). Error bars depict the standard error of the mean ΔCt values. Significant differences (*P* < 0.05) are denoted with a star. **D.** Western blot analysis showing the down-regulation of FA genes in PAK1 depleted cells. *PAK1* amplified and non-amplified breast cancer cells were transfected with non-targeting or *PAK1* targeting siRNAs, total protein was extracted and western blots were performed using the indicated antibodies.

To identify differentially regulated genes between wild-type and PAK1 deficient mouse and human breast cancer cells, we extracted total RNA from the genetically engineered human cell line MCF10A.B2 expressing an inducible shRNA against PAK1, and from *PAK1−/−* breast cancer cell lines derived from murine tumors [14], and performed a comparative gene profiling study by using human or mouse whole genome arrays. A considerable number of differentially expressed genes between wild-type and PAK1 deficient human breast cancer cells were also found differentially expressed in mouse breast cancer cells (Figure 1A). Interestingly, several genes involved in the FA/BRCA pathway, a DNA-damage response signaling pathway which is essential for the repair of DNA interstrand cross-links induced by DNA-damaging agents like cisplatin and doxorubicin [15, 16], were down-regulated in PAK1 deficient cells (Figure 1B). To test if the down-regulation of the aforementioned genes correlated with *PAK1* expression levels, the total amount of PAK1 and its phosphorylation levels were confirmed by western blot in the *PAK1* non-amplified breast cancer cell line HCC1419, the PAK1 overexpressing cell lines BT-474 and MDA-MB-361 and the *PAK1* amplified breast cancer cell line SK-BR-3 (Figure S1A). Next, the expression level of two FA/BRCA genes, FANCD2 and FANCI, was confirmed by qPCR and western blot in PAK1 depleted human breast cancer cells (Figure 1C and 1D). Interestingly, the absence of *PAK1* drastically affected the expression of the FA/BRCA genes in the breast cancer cell lines with amplification and/or overexpression of *PAK1* and had little effect in breast cancer cells with low expression levels of this protein kinase. These findings are consistent with a recent study were a TCGA analysis showed that *PAK1* overexpression was correlated with the expression of *BRCA1* and *BRCA2* in inflammatory breast cancer [17]. Finally, the results of a TCGA analysis we performed showed that *PAK1* overexpression in human breast cancer samples correlates with the expression of most *FA/BRCA* genes, particularly with *FANCI* (Figure S1B).

### PAK1 depletion or inhibition sensitizes 11q13 amplified breast cancer cells to DNA damaging agents

**Figure 2:**
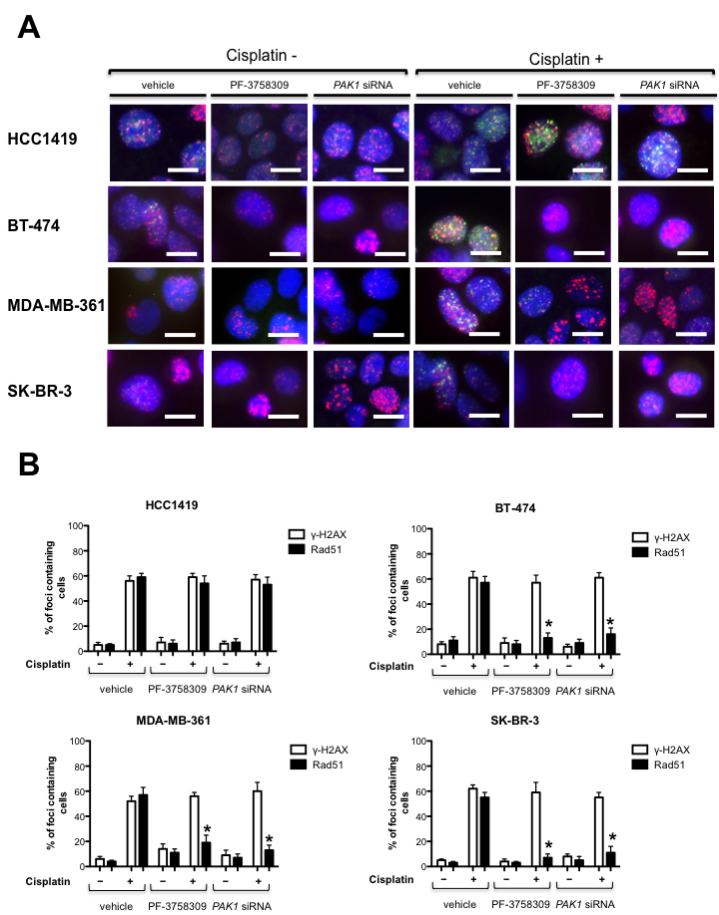
PAK inhibition reduces the formation of Rad51 foci **A.** PAK inhibition affect DNA repair by homologous recombination in PAK1 overexpressing and/or *PAK1*-amplified breast cancer cells, but not in non-amplified brest cancer cells. HCC1419, BT-474, MDA-MB-361 and SK-BR-3 cells were treated with vehicle, 1 μM of the PAK inhibitor PF-3758309 or transfected with *PAK1* targeting siRNAs, and incubated 24 h with or without 10 μM cisplatin, fixed and stained with anti Rad51 (green), anti γ-H2AX (red) and DAPI (blue). The data are representative of 3 independent experiments. **B.** The graphics show the percent ± SD of cells containing ≥5 Rad51 and γ-H2AX foci. The data are representative of 3 independent experiments. Bars ± SD. * *P* > 0.05, ** *P* > 0.001.

Since FA/BRCA deficient cells are defective in the formation of Rad51 foci, which is a crucial component of the HR repair machinery [11, 18], we examined the effect of PAK inhibition in the ability of FA/BRCA proficient cells to form these foci. To this end, the breast cancer cell lines with or without *PAK1* amplification and/or overexpression, were treated with vehicle or PF-3758309, a small molecule inhibitor of Group A and Group B PAKs [19], or transfected with *PAK1* targeting siRNAs, and DNA damage was induced with cisplatin. Interestingly, we found that PAK inhibition or depletion in *PAK1* amplified or overexpressing breast cancer cells significantly reduced the formation of Rad51 foci, but had no effect in *PAK1* non-amplified breast cancer cells which were able to form Rad51 foci in response to DNA damaging agents (Figure 2A and 2B). These data strongly suggest that PAK inhibition affects DNA repair by HR only in FA/BRCA proficient breast cancer cells with amplification or overexpression of *PAK1.*

### PAK1 depletion or inhibition reduces cell survival

**Figure 3:**
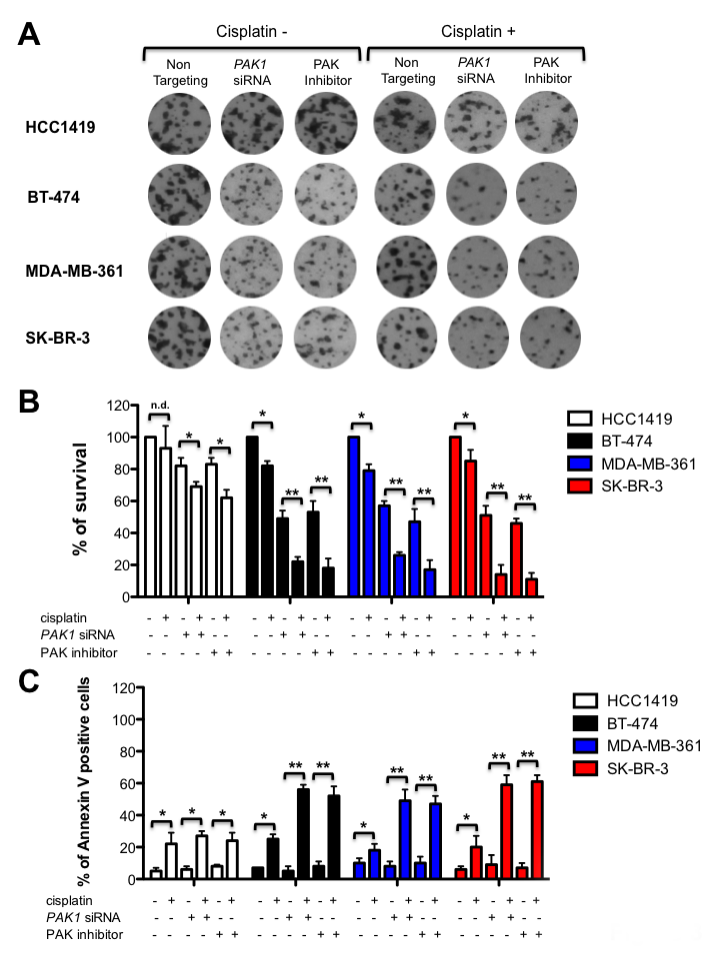
PAK inhibition reduces cell survival and promotes apoptosis **A.** PAK inhibition sensitizes *PAK1* amplified breast cancer cells to cisplatin treatment. HCC1419, BT-474, MDA-MB-361 and SK-BR-3 cells were assessed for colony formation after vehicle or PF-3758309 treatment, or transfection with *PAK1* targeting siRNAs in the presence or absence of cisplatin. Representative plates of three different experiments are shown. **B.** Mean survival is graphed after vehicle or PF-3758309 treatment, or transfection with *PAK1* targeting siRNAs plus or minus cisplatin, expressed as a percentage of colonies formed ± SD compared to vehicle-treated cells. **C.** PAK inhibition and cisplatin treatment promotes apoptosis of PAK1-overexpressing and/or amplified breast cancer cells. Cells were treated as described in 3A for 4 days, collected, and apoptosis was measured calculating the percent of positive Annexin V-phycoerythrin cells by flow cytometry. The data are representative of 3 independent experiments. Bars ± SD. * *P* > 0.05, ** *P* > 0.001.

Next, we examined if PAK inhibition impacts cell survival in long-term colony formation assays. HCC1419, BT-474, MDA-MB-361 and SK-BR-3 breast cancer cells were treated with vehicle or PF-3758309, or transfected with *PAK1* targeting siRNAs and exposed to cisplatin. As expected, PAK inhibition had little effect on the survival of HCC1419 cells, even in the presence of cisplatin. However, PAK depletion or inhibition caused more than 50% reduction in the survival of BT-474, MDA-MB-361 and SK-BR-3 cells treated with cisplatin (Figure 3A and 3B). To determine whether PAK inhibition could promote cell death in these cells, we calculated the percentage of apoptotic cells in HCC1419, BT-474, MDA-MB-361 and SK-BR-3 breast cancer cells treated with vehicle, PF-3758309 or transfected with *PAK1* targeting siRNAs and incubated with cisplatin. Consistent with our previous results, we found that breast cancer cells with amplification or overexpression of *PAK1* are highly sensitive to cisplatin-induced apoptosis after *PAK1* inhibition or depletion, whereas PAK blockade has a very modest effect in HCC1419 breast cancer cells (Figure 3C).

### Reduced PAK1 activity sensitizes breast cancer cells to PARP inhibition

Since FA/BRCA deficient cells and other cells that are deficient in HR are highly susceptible to PARP small molecule inhibitors [11, 20, 21], we hypothesized that PAK inhibition could sensitize *PAK1* amplified or overexpressing beast cancer cells to PARP pharmacological inhibition. To this end we tested the effect of small molecule inhibitors of PAK and PARP, alone and together, on Rad51 foci formation in breast cancer cells with amplification or overexpression of *PAK1.* These compounds included PF-3758309 and Rucaparib, which is a potent inhibitor of PARP-1 and PARP-2 [22].

**Figure 4:**
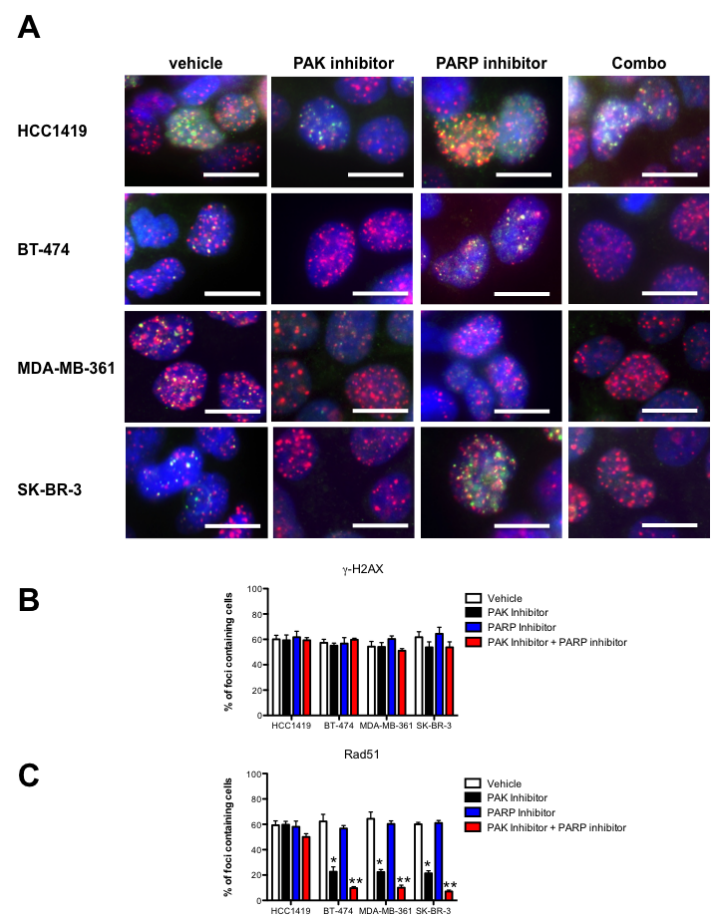
Combined PAK and PARP inhibition impairs DNA repair by Homologous Recombination in PAK overexpressing breast cancer cells SK-BR-3, BT-474, MDA-MB-361 and HCC1419 cells were treated with vehicle, 1 μM of the PAK inhibitor PF-3758309 and/or 1 μM of rucaparib, and incubated 24 h with 10 μM cisplatin, fixed and stained with anti Rad51 (green), anti γ-H2AX (red) and DAPI (blue). B, The graphics show the percent ± SD of cells containing 10 Rad51 and γ-H2AX foci. The data are representative of 3 independent experiments. Bars ± SD. * *P* > 0.05, ** *P* > 0.001

**Figure 5:**
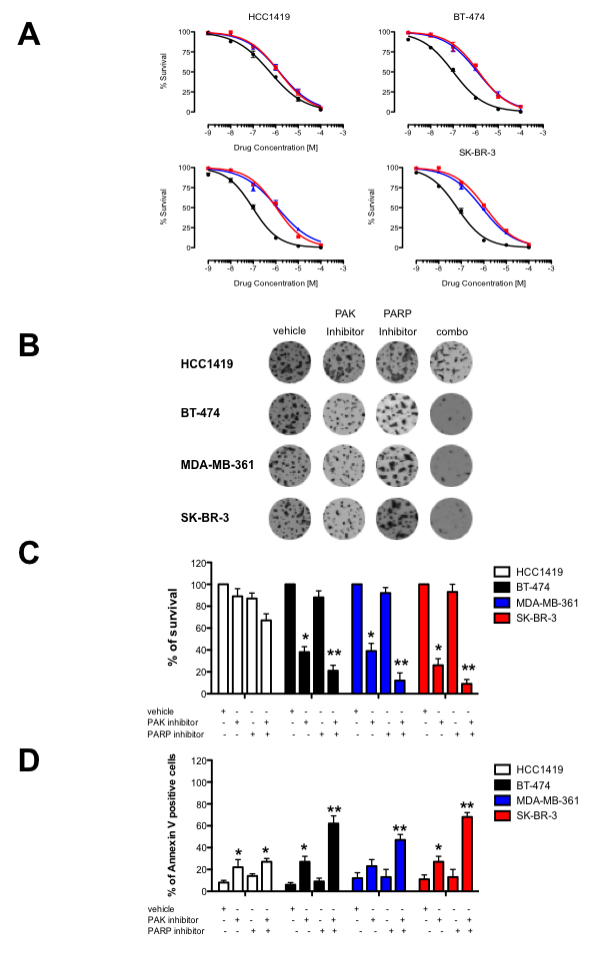
Combined PAK and PARP inhibition have a synergistic effect *in vitro* **A.** Effect of PAK and PARP inhibitors on survival of PAK1-overexpressing breast cancer cells. HCC1419, BT-474, MDA-MB-361 and SK-BR-3 cells were treated with the indicated amounts of PF-3758309 (red lines), rucaparib (blue lines) or both drugs (black lines) for 4 days; cell viability was determined by Trypan blue exclusion. **B.** Combination of PF-3758309 and/or rucaparib treatment decreases cell survival of PAK1 overexpressing breast cancer cells. Cells were assessed for colony formation after vehicle, PF-3758309 and/or rucaparib treatment. Mean survival from three experiments is expressed as a percentage of colonies formed ± SE relative to vehicle-treated cells. Representative plates are shown and mean survival is graphed after vehicle, PF-3758309 and/or rucaparib exposure, expressed as a percentage of colonies formed ± SD compared to vehicle-treated cells. **C.** Combination of PF-3758309 and/or rucaparib treatment promotes apoptosis of *PAK1* amplified and/or overexpressing breast cancer cells. Cells were treated with the indicated amounts of PF-3758309 and/or rucaparib for 4 days, collected, and apoptosis was measured calculating the percent of positive Annexin V-phycoerythrin cells by flow cytometry. The data are representative of 3 independent experiments. Bars ± SD. * *P* > 0.05, ** *P* > 0.001

SK-BR-3, BT-474, MDA-MB-361 (*PAK1* amplified and/or overexpressing cells) and HCC1419 cells were treated with these inhibitors and cisplatin, and the effect on Rad 51 foci formation was assessed following 3 days of treatment. As expected, there are no differences on the γ-H2AX foci number among all the cell lines tested, independently of PAK1 expression levels. However, PAK inhibition significantly affected the formation of Rad51 foci only in *PAK1-*amplified and/or overexpressing cells, and this effect is much more drastic in cells treated with both, PAK and PARP inhibitors and exposed to cisplatin (Figure 4). To test the effect of dual inhibition of PAK and PARP in the survival of breast cancer cells, we performed a synergy test (Figure 5A and Table 1). The PARP inhibitor alone had a nearly identical effect in the survival of all breast cancer cell lines, and as expected, PAK1 overexpressing and *PAK1*-amplified breast cancer cells were much more sensitive to PAK inhibition than HCC1419 cells. The IC50 values for Rucaparib in BT-474, MDA-MB-361, SK-BR3 and HCC1419 cells were 101.46, 98.8, 98.1 and 102.3 nmol/L respectively; and the IC50 values for PF-3758309 were 74.3, 66.8, 65.6 and 97.0 nmol/L respectively. However, when the compounds were coadministered, a marked synergistic effect was noted only in PAK1 overexpressing and *PAK1*-amplified breast cancer cells [combination index (CI) < 0.5; Figure 5A and Table 1]. Coadministration of PF-3758309 and Rucaparib yielded CI values of 19.8, 21.5 and 19.3 nmol/L in BT-474, MDA-MB-361 and SK-BR-3 cells, indicating a high degree of synergy, whereas this effect was not seen in HCC1419 cells (Figure 5A). Next, we examined if combined PAK and PARP inhibition affects cell survival in colony formation assays. Treatment of HCC1419 cells with each of these drugs reduced approximately 20% the number of colonies, and the coadministration of both compounds caused a 35% reduction in the number of colonies. Treatment of PAK1 overexpressing and *PAK1*-amplified breast cancer cells with each of these drugs had a similar effect, and interestingly, coadministration of both inhibitors caused a very significant reduction (72-78 %) in cell survival (Figure 5B and 5C). In addition, the combination of PAK and PARP-targeting agents, did not merely produce cytostasis, but also resulted in cell death; increasing the frequency of apoptosis in PAK1 overexpressing and *PAK1*-amplified breast cancer cells by nearly a factor of 3 (Figure 5D).

In order to demonstrate that these effects were dependent of PAK1 expression levels, HCC1419 cells were transfected with a vector encoding a myc-tagged wild type PAK1 (Figure S2A), and the effect of small molecule inhibitors of PAK and PARP on Rad51 foci formation was tested in cisplatin treated cells. Our results showed that the pharmacological inhibition of PAK alone or in combination with PARP in the myc-PAK1 overexpressing cells reduced their ability to form Rad51 foci in response to DNA damaging agents (Figure S2B), suggesting that the inhibition of this kinase in PAK1 overexpressing cells sensitize them to PARP pharmacological inhibition and has a negative impact in DNA repair by HR. In addition, survival and apoptosis of the myc-PAK1 overexpressing cells were tested as previously described. Interestingly, the colony formation ability of these cells was drastically reduced after PAK and PARP pharmacological inhibition, and also resulted in cell death (Figure S3).

**Table 1:** Synergistic effect of PAK and PARP inhibitors

				CI (average ± std. dev)		
Cell line	Inhibitors		Molar ratio	ED50	ED75	ED95
**HCC1419**	**PAK inhibitor**	**PARP inhibitor**	**1:1**	**0.858 ± 0.085**	**0.963 ± 0.126**	**1.026 ± 0.065**
**MDA-MB-436**	**PAK inhibitor**	**PARP inhibitor**	**1:1**	**0.426 ± 0.103**	**0.483 ± 0.116**	**0.603 ± 0.092**
**BT-474**	**PAK inhibitor**	**PARP inhibitor**	**1:1**	**0.418 ± 0.078**	**0.502 ±0.104**	**0.598 ± 0. 133**
**SK-BR-3**	**PAK inhibitor**	**PARP inhibitor**	**1:1**	**0.402 ± 0.036**	**0.496 ± 0.122**	**0.516 ± 0.042**

Summary results of drug interactions calculated as Chou-Talalay CI based on CellTiter-Blue viability determinations. CI values <0.5 (underlined) indicated strong synergy between the two small molecule inhibitors in producing cytotoxic effect.

### Pharmacological inhibition of PAK and PARP impairs tumor growth *in vivo*

We next tested the effects of these small molecule inhibitors on the growth of SK-BR-3 xenografts. SK-BR-3 cells were xenografted to severe combined immunodeficient (SCID) mice and tumors were allowed to form for 7 days. The mice were then treated with vehicle, PAK inhibitor PF-3758309, PARP inhibitor Rucaparib, or PF-3758309 plus Rucaparib, for 15 days. Tumor volumes were assessed every 3 days, and the animals were sacrificed at the end of the treatment.

**Figure 6:**
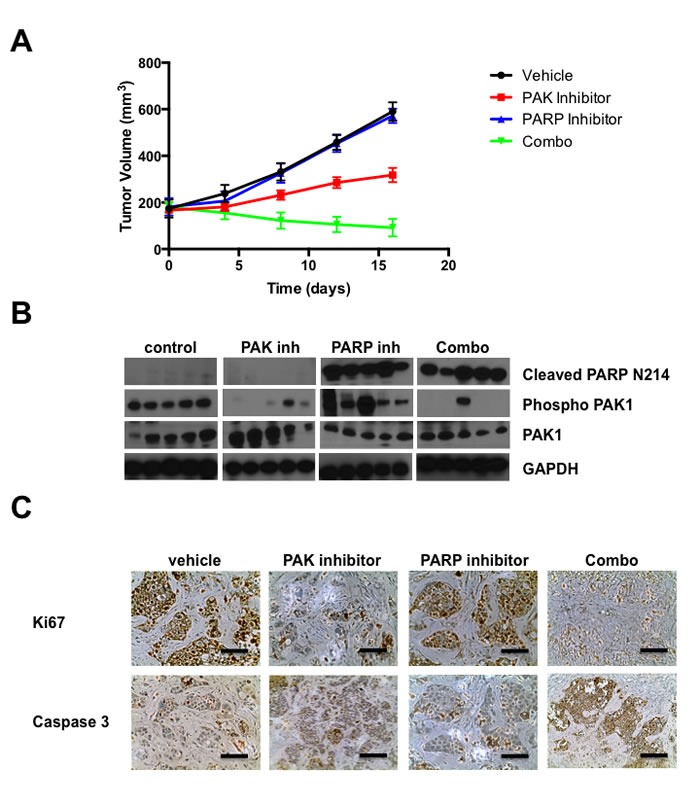
Pharmacological inhibition of PAK and PARP impairs tumor growth *in vivo* Inhibition of PAK and PARP impedes the tumorigenicity of breast cancer cells. SK-BR-3 cells were injected into the mammary glands of C. B17/IcrSCID mice. Ten days after innoculation, the animals were treated with vehicle or PAK and/or PARP inhibitors for 15 days. **A.** Volumetric changes in tumor size between untreated mice (vehicle) and mice treated with inhibitors. **B.** Representative western blots showing PAK and PARP activity in tumors dissected from mice treated with vehicle or PAK and/or PARP inhibitors. **C.** Representative example of tumor sections between untreated mice and mice treated with PAK inhibitor, PARP inhibitor and a combination of PAK and PARP inhibitors stained for cleaved caspase-3 and Ki67.

Treatment with PF-3758309 had a marked negative effect on tumor growth, yielding tumors of about one-half the volume of tumors in untreated animals. Rucaparib alone did not affected tumor growth. Interestingly, animals treated with the combined PAK and PARP inhibitors showed no tumor growth (Figure 6A).

Analysis of markers of cell proliferation and apoptosis revealed that PF-3758309 treatment prevented cell proliferation and induced apoptosis, whereas Rucaparib did not affect proliferation but slightly increased apoptosis. When coadministered, the inhibitors blocked proliferation and caused extensive apoptosis (Figure 6B).

## DISCUSSION

The chromosomal region 11q13 is amplified in approximately 15-20 of breast cancers and has been associated with the presence of lymph node metastases, poor prognosis and lower survival rates [23-25]. A number of potential oncogenes have been mapped in this region, which have been suggested to play roles in the genesis and maintenance of breast cancer [26, 27].

The PAK1 gene, which resides in 11q13.5, has previously been implicated in breast cancers and other cancers that contain this amplicon. It has been shown that PAK1 gene is frequently amplified in human breast cancer; PAK1 amplification is associated with resistance to tamoxifen; transgenic expression of an activated PAK1 allele induces transformation of mammary epithelial cells in culture and induces breast cancer in mice; and expression of dominant-negative alleles, shRNAs, or treatment with PAK inhibitors, impede the growth and/or normalize the morphology of various breast cancer cell lines in tissue culture [28-32]. However, none of these studies examined the effect of PAK1 inhibition in the genetic profile of breast cancer cells, nor did they use clinically relevant small molecule inhibitors. In this study, we show that (a) blockade of PAK1 expression or activity *in vitro* down-regulates the expression of FA/BRCA pathway genes, (b) knock down or pharmacological inhibition of PAK in PAK1 overexpressing cells, compromises the ability of these cells to form Rad51 foci, (c) loss of PAK function reduces cell survival and promotes apoptosis, (d) PAK inhibition in PAK1 overexpressing breast cancer cells, sensitize these cells to PARP inhibition, and (e) small molecule inhibitors of PAK and PARP have a synergistic effect *in vitro*, and impair tumor growth in a xenograft setting.

The molecular mechanism by which PAK influences the expression of FA/BRCA genes is unknown. However, recent studies have demonstrated that all the genes of the FA/BRCA family posses a highly conserved promoter region [33], which contains DNA binding sites for transcription factors that are phosphorylated and activated by PAK1. This is the case of NF-κB, which is activated by PAK1, allowing the translocation of the p65 subunit into the nucleus where it acts as a transcription factor [34]. Another studies have also shown that even when PAK1 cannot bind directly to the DNA, it can form part of transcriptionally active complexes [5, 13, 35], suggesting that PAK1 could promote the transcription of FA/BRCA genes directly or indirectly. In addition, a recent study has shown that *PAK1* overexpression correlates with the expression of *FA/BRCA* genes in inflammatory breast cancer samples [17]. Finally, our results of a TCGA analysis showed that *PAK1* overexpression in human breast cancer specimens correlates with the expression of most *FA/BRCA* genes.

Interestingly, we observed that depletion or pharmacological inhibition of PAK in PAK1 overexpressing breast cancer cells, drastically reduced the expression of FANCI and FANCD2, cell survival and the ability of these cells to form Rad51 foci in response to DNA damaging agents, but in contrast, PAK inhibition only had a modest effect in these cellular processes in breast cancer cells that express low levels of PAK1. These findings are consistent with a previous report showing a strong correlation between PAK1 expression and the expression of proteins involved in DNA damage response in Primary Esophageal Small Cell Carcinoma (PESCC) [36], suggesting that PAK1 may be an important player in this cellular process.

Remarkably, we found that PAK inhibition sensitizes PAK1 overexpressing breast cancer cells to PARP inhibition. It is well documented that FA/BRCA-defective cells and other cells defective in HR are highly susceptible to PARP small molecule inhibitors [11]. Therefore, the down-regulation of FA/BRCA genes mediated by PAK1 creates a state of “FA/BRCAness”, and represents a rational approach for expanding the efficacy of PARP inhibitors to FA/BRCA-proficient cancer populations.

## MATERIALS AND METHODS

### Cell culture, expression plasmids and transfection

The mouse tumor derived breast cancer cell lines Neu:PAK1^+/+^ and Neu:PAK1^−/−^ were maintained in low calcium medium supplemented with 5% horse serum, 50 U/mL penicillin, and 50 mg/mL streptomycin as previously described [14]. Wild type 10A.ErbB2 cells (MCF-10A cells expressing a chimeric form of ErbB2) and 10A.ErbB2 cells expressing a tetracycline inducible shRNA against PAK1 (described in [14]) were maintained in DMEM/F12 (Gibco BRL) supplemented with 5% donor horse serum, 20 ng/mL EGF (Harlan Bioproducts), 10 mg/mL insulin (Sigma), 1 ng/mL cholera toxin (Sigma), 100 mg/mL hydrocortisone (Sigma), 50 U/mL penicillin, and 50 mg/mL streptomycin [37]. HCC1419, MDA-MB-361, BT-474, and SK-BR3 were obtained from American Type Culture Collection, HCC1419 and MDA-MB-361cells were grown in RPMI-1640 supplemented with 10% FBS, BT-474 cells were grown in DMEM/F12 supplemented with 10% FBS and SK-BR3 were grown in McCoy's 5A supplemented with 10% FBS.

For transient transfection experiments, the pCMV6M-PAK1 vector was transfected into HCC1419 cells by using Lipofectamine 2000 (Invitrogene).

### Microarrays

RNA from the mouse tumor derived breast cancer cell lines Neu:PAK1^+/+^ and Neu:PAK1^−/−^, and from the human MCF10A.B2 and MCF10A.B2 cells expressing a shRNA against PAK1, was purified from whole-cell lysates using the RNeasy mini kit (Qiagen), and contaminating DNA was removed using a RNase-free DNase set. A quantity amounting to 500 ng total RNA was amplified and labeled using the low RNA input linear amplification kit (Agilent). Labeled cRNA targets were hybridized onto human or mouse whole genome arrays. Microarray images were processed using Agilent Feature Extraction software (version 9.5). Data were background corrected using the *normexp* method (PMID: 17720982) implemented in the Bioconductor package *limma*, and quantile normalized. Identification of differentially expressed genes was performed with empirical Bayes moderated *t* tests using *limma*. Biological pathways and networks were examined with Ingenuity Pathway Analysis software (www.ingenuity.com). The microarray original data have been submitted to Gene Expression Omnibus (GEO) database (Accession number: GSE72206).

### Real-time PCR

Total RNA was extracted from cells using RNeasy Mini kits, quantified by Nanodrop ND-1000 and reverse transcribed using the High Capacity cDNA Reverse Transcription Kit (Applied Biosystems). 1 ng cDNA was amplified by real time PCR using Universal ProbeLibrary (UPL) probes (Roche). The sequences for the primers for real-time qPCR were: FANCI Fwd: 5′, Rev: 5′ 3′; FANCD2 Fwd; 5′: 3′, Rev: 5′ 3′; PAK1 Fwd: 5′ 3′, Rev: 5′ 3′; and GAPDH Fwd: 5′ 3′. UPL probes used were #80 for FANCI, #69 for FANCD2, #19 for PAK1 and #63 for GAPDH. Each sample was run in 20 μl reaction using 2X FastStart Universal Probe Master with ROX (Roche). Reactions were performed in an ABI real time PCR 7500 (Applied Biosystems, Foster City, CA). Ratios of mRNA levels to control values were calculated using the ΔCt method (2^−ΔΔCt^) at a threshold of 0.02 [38]. All data were normalized to control GAPDH. PCR conditions used: hold for 10 min at 95°C, followed by 40 cycles of 15 s at 95°C and 60 s at 60°C.

### Immunofluorescence and confocal microscopy

PAK1 amplified, overexpressing and non-amplified breast cancer cells were grown on cover slips in the presence of cisplatin and/or the PAK inhibitor PF-3758309 and/or the PARP inhibitor rucaparib. Cells were then fixed with 4% paraformaldehyde for 30 min, washed three times with PBS, permeabilized with 0.5% Triton X-100 for 10 min and blocked with 1% albumin in PBS for 30 min at room temperature. Cells were incubated overnight with antibodies specific for -H2AX (pSer139) (Upstate Biotechnology 05-636, clone JBW301), and Rad51 (Santa Cruz Biotechnology sc8349, clone H-92), washed three times with PBS and incubated with Alexa Fluor 488 conjugated secondary antibodies (Life Technologies). The nucleus were counterstained with DAPI (4′,6′-diamidino-2-phenylindole). Confocal analyses were performed with a Nikon TE2000 confocal microscopy system and the number of foci per cell was calculated by dividing the total number of foci in the frame to the number of cells containing foci.

### Colony formation assays

For colony formation assays, SK-BR3 (PAK1 amplified), BT-474 and MDA-MB-361 (PAK overexpressing) and HCC1419 (non-amplified) cells were seeded in six-well plates at 1,000 cells per well in the presence of cisplatin and/or the PAK inhibitor PF-3758309 and/or the PARP inhibitor rucaparib. Colony formation was assessed 2 weeks after plating with crystal violet staining. For siRNA treatments, exponentially growing cells were reverse-transfected with Dharmacon siRNAs targeting PAK1 in 24-well plates, and 2 d post-transfection cells were treated with cisplatin or rucaparib and then replated in six-well plates for colony formation. Mean colony formation from three experiments was expressed as percentage of colonies ± SE relative to vehicle-treated cells.

### Apoptosis analyses

Apoptosis was measured using the Annexin V-PE Apoptosis Detection kit (BD Pharmingen) followed by flow cytometry. Amplified, overexpressing and non-amplified PAK1 breast cancer cells (2 × 10^5^) were seeded in six-well cell culture plates and treated with vehicle, cisplatin and/or the PAK inhibitor PF-3758309. Both floating and attached cells were collected 4 d after cell seeding, washed twice with cold PBS, and suspended in 1× binding buffer. A 100 μL aliquot of the cell suspension (representing 5 × 10^4^ cells) was transferred to a culture tube, to which 5 μL of Annexin V-PE and 5 μL of 7- aminoactinomycin D (7-AAD) were added, and the mix was incubated for 15 min at room temperature in the dark. Apoptosis analysis was carried out using a FACScan and FlowJo software version 7.2. A total of 10,000 cells were collected for each sample for analysis.

### Drug synergy testing

The combination index (CI) between pharmacological inhibitors was established by the Chou-Talalay method [39]. We used the software package CalcuSyn (BioSoft, UK) to automate calculations. Briefly, for each drug tested, an IC50 curve was established in each cell line, and used to select combination doses of drugs for subsequent synergy tests. 3500 cells were plated per well in 96-well plates. After 24 hours, cells were treated with serial dilutions of individual inhibitors or combinations of two inhibitors maintained at a constant molar ratio. After 72 hours incubation, cell viability was measured using either CellTiter Blue (Promega, USA) or a WST1 assay (Roche Applied Science, Indianapolis, IN). The CI values for each dose and corresponding cytotoxicity were expressed as the fraction affected (Fa) and were calculated using CalcuSyn computer software and presented as Fa-CI plots.

### Tissue preparation, histology, immuno-histochemistry, and immunoblotting

All tumor samples and control tissues were fixed overnight in 4% paraformaldehyde, dehydrated, and embedded in paraffin. Hematoxylin and eosin (H&E)-stained sections were used for diagnostic purposes and unstained sections for immunohistochemical studies. Protein concentration was determined, and equal amounts of total proteins were separated on SDS- PAGE. IHC was performed with the following antibodies: rabbit polyclonal antibody for Cleaved Caspase-3 (Cell Signaling) and Ki-67 (Santa Cruz Biotechnology). The evaluation of the IHC was conducted blindly, without knowledge of the treatment. Immunoblot analyses were performed on lysates extracted from tumors. Antibodies used for western blot included PAK1, phospho-PAK1, cleaved caspase-3, H2AX and phospho-H2AX from Cell Signaling Technology; Rad51, FANCI, FANCD2 and Ki-67 were from Santa Cruz Biotechnology. GAPDH was used as loading control.

### Tumor xenografts

Four- to 6-week-old inbred C.B17/Icr-SCID mice were obtained from the Jackson Labs. SK-BR-3 cells (5 × 10^6^ in 0.3 ml of rBM) were injected into the mammary fat pad of each mouse. Mice were treated with either vehicle or PAK inhibitor PF-3758309 at dose of 20 mg/kg/day, PARP inhibitor at dose of 50 mg/kg/day, in the combination groups, the compounds were given with 4-6 h interval. At completion of all xenograft studies mice were sacrificed, the tumors were excised and tumor volumes estimated with the following formula: volume = (a2 X b) / 2, where a = short and b = long tumor lengths, respectively, in millimeters.

### Statistical analysis

Statistical analysis was conducted using the unpaired Student t test except for survival curves where the log *P* rank test was used. Values of *P* < 0.05 were considered significant

## SUPPLEMENTARY MATERIALS FIGURES


