# Supplementary material for: Reduced PAK1 activity sensitizes FA/BRCA-proficient breast cancer cells to PARP inhibition
Source: Oncotarget. 2016 Oct 11;7(47):76590–603. doi: 10.18632/oncotarget.12576 (PMC5363532; doi:10.18632/oncotarget.12576)
Supplement: Supplementary file 1 [file oncotarget-07-76590-s001.pdf]

Reduced PAK1 activity sensitizes FA/BRCA-proficient breast cancer cells to PARP inhibition

Supplemental Information

1. Supplemental Data

Figure S1, related to Figure 1.

Figure S2, related to Figure 4.

Figure S3, related to Figure 5.

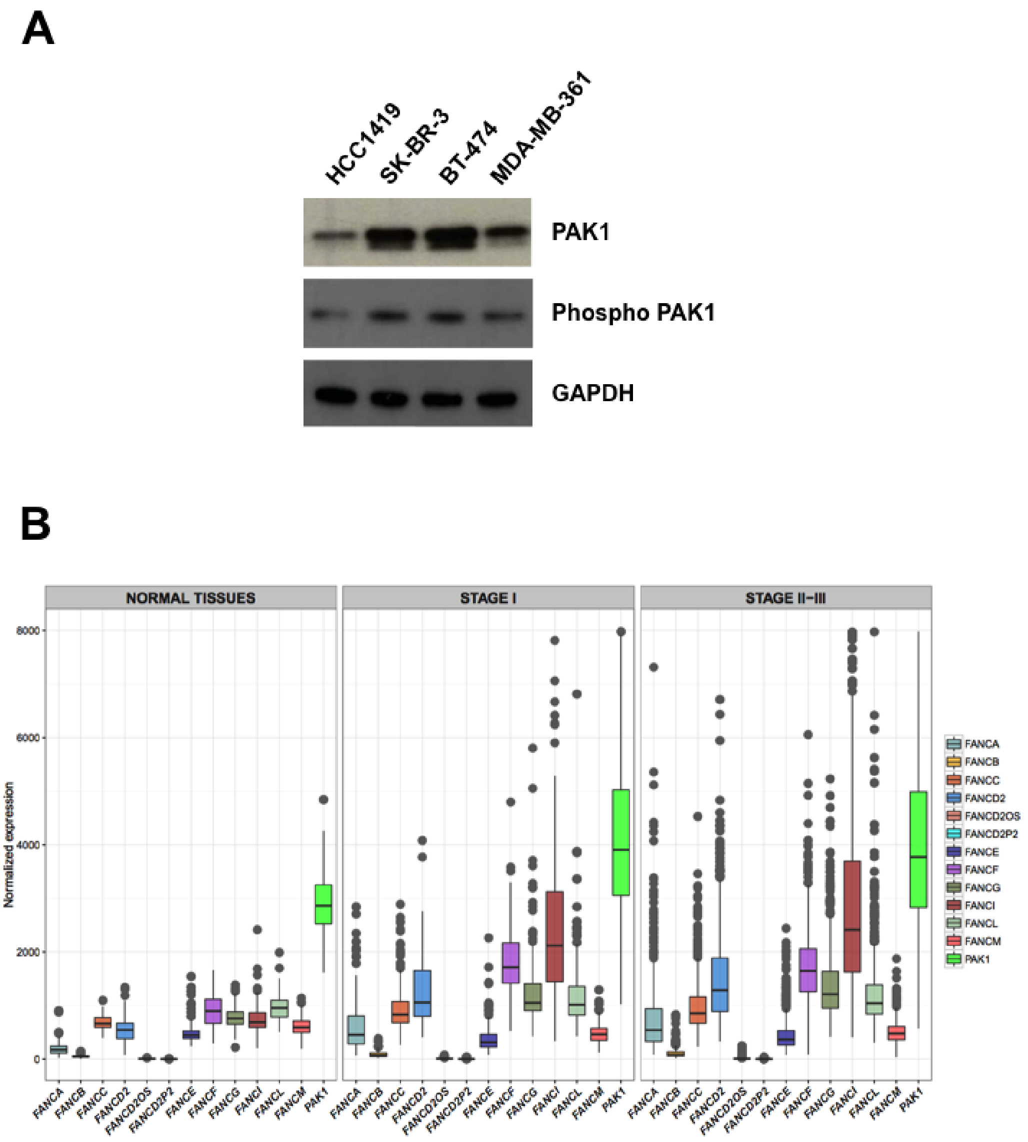

Figure S1. *PAK1* and *FA/BRCA* related genes expression levels across breast cancer tissues. A, Representative western blots showing total PAK1 expression levels and PAK1 activity in the breast cancer cell lines HCC1419, BT474, SK-BR-3 and MDA-MB-361, GAPDH was used as loading control. B, Normalized gene expression values from 113; 181 and 886 normal breast tissues; stage I and stage II-III, respectively were downloaded from TCGA RNAseq V2 and graphed as relative total counts.

A

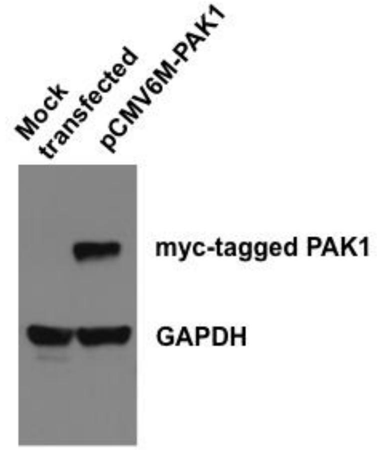

B

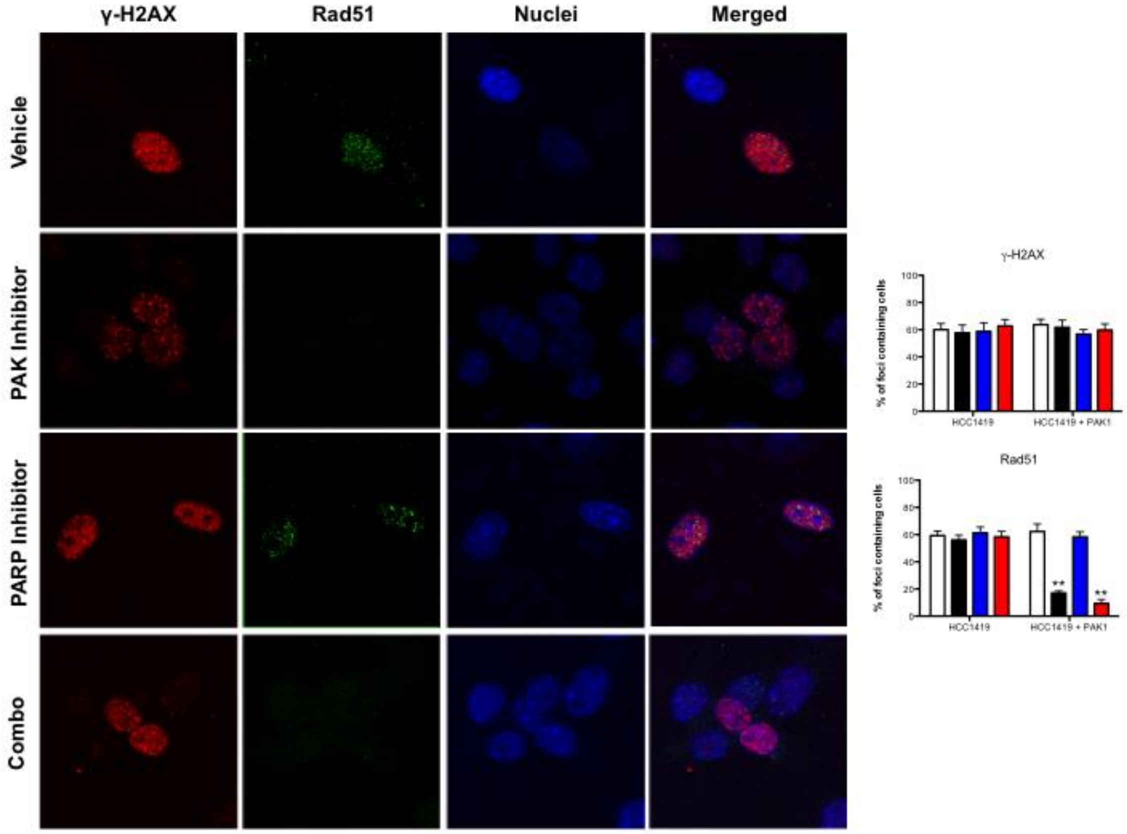

Supplemental Figure S2. Combined PAK and PARP inhibition impairs Rad51 foci formation in breast cancer cells that ectopically express wild type PAK1. A, Ectopic expression of myc-tagged wild type PAK1 in HCC1419

breast cancer cells. HCC1419 cells were mock transfected or transfected with a vector encoding my-tagged wild type PAK1. The expression of myc-PAK1 was assessed by western blot using an anti-myc antibody, GAPDH was used as loading control. B, HCC1419 cells ectopically expressing myc-tagged PAK1 were treated with vehicle, 1 μM of the PAK inhibitor PF-3758309 and/or 1 μM of rucaparib, and incubated 24 h with 10 μM cisplatin, fixed and stained with anti Rad51, anti γ-H2AX and DAPI. The graphics show the percent ± SD of cells containing 10 Rad51 and γ-H2AX foci. Empty boxes represent cells treated with vehicle, black boxes cells treated with PAK inhibitor, blue boxes cells treated with PARP inhibitor and red boxes cells treated with both PAK and PARP inhibitors. The data are representative of 3 independent experiments. Bars ± SD. * $P > 0.05$, ** $P > 0.001$

A

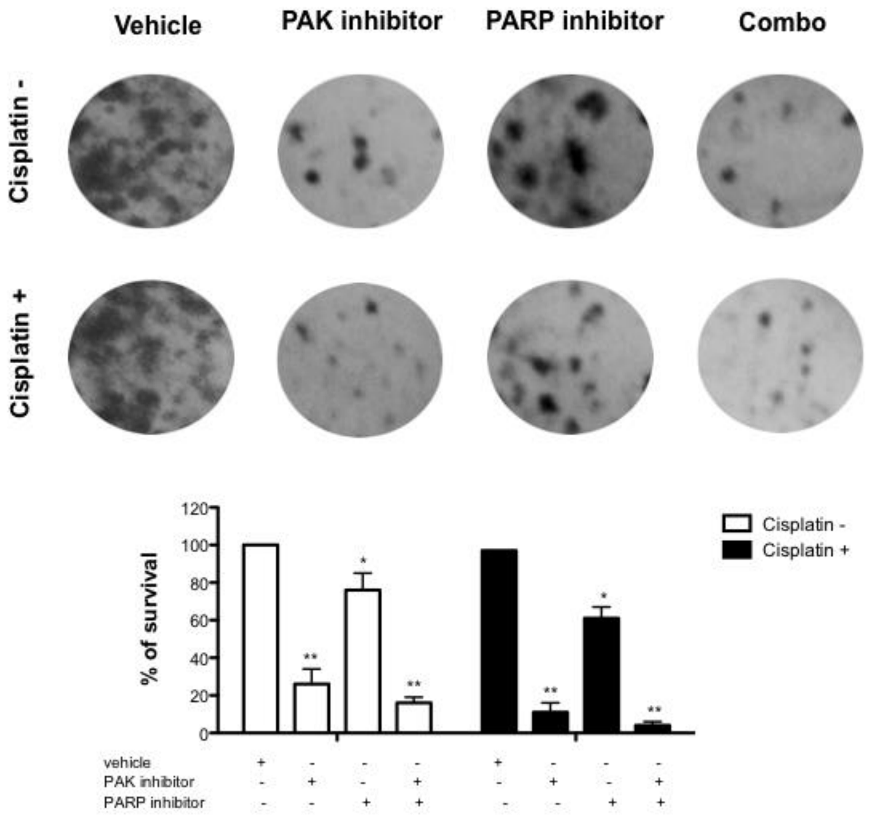

B

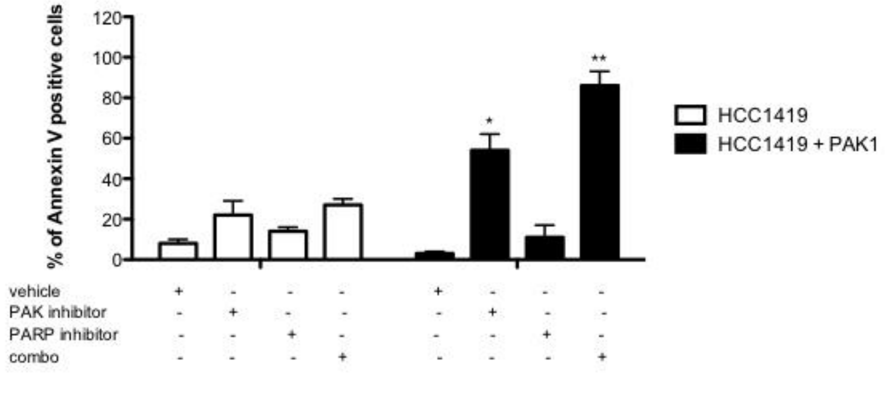

Supplemetal Figure S3. Combined PAK and PARP inhibition decreases cell survival of HCC1419 cells ectopically overexpressing PAK1. A, Combination of PF-3758309 and/or rucaparib treatment decreases cell survival of HCC1419

cells ectopically overexpressing PAK1. Cells were assessed for colony formation after vehicle, PF-3758309 and/or rucaparib treatment. Mean survival from three experiments is expressed as a percentage of colonies formed ± SE relative to vehicle-treated cells. Representative plates are shown and mean survival is graphed after vehicle, PF-3758309 and/or rucaparib exposure, expressed as a percentage of colonies formed ± SD compared to vehicle-treated cells. B, Combination of PF-3758309 and/or rucaparib treatment promotes apoptosis of HCC1419 cells ectopically overexpressing PAK1. Cells were treated with the indicated amounts of PF-3758309 and/or rucaparib for 4 days, collected, and apoptosis was measured calculating the percent of positive Annexin V-phycoerythrin cells by flow cytometry. The data are representative of 3 independent experiments. Bars ± SD. * $P > 0.05$, ** $P > 0.001$
